# Operational lessons learned in conducting an international study on pharmacovigilance in pregnancy in resource-constrained settings: The WHO Global Vaccine safety Multi-Country collaboration project

**DOI:** 10.1016/j.jvacx.2022.100160

**Published:** 2022-04-09

**Authors:** Apoorva Sharan, Shubhashri Jahagirdar, Anke L Stuurman, Varalakshmi Elango, Margarita Riera-Montes, Neeraj Kumar Kashyap, Narendra Kumar Arora, Mathews Mathai, Punam Mangtani, Hugo Devlieger, Steven Anderson, Barbee Whitaker, Hui-Lee Wong, Clare L Cutland, Christine Guillard Maure, the WHO Global Vaccine Safety Multi-Country Collaboration Sites

**Affiliations:** a The INCLEN Trust International, New Delhi, India; b Swiss Tropical and Public Health Institute (Swiss TPH), Basel, Switzerland; c University of Basel, Basel, Switzerland; d P95 Pharmacovigilance and Epidemiology, Leuven, Belgium; e Centre for Maternal and Newborn Health, Liverpool School of Tropical Medicine, Liverpool, UK; f Department of Infectious Disease Epidemiology, London School of Tropical Medicine, London, UK; g Universitair Ziekenhuis, Leuven, Belgium; h Center for Biologics Evaluation and Research (CBER), U.S. Food and Drug Administration (FDA), Silver Spring, MD, USA; i African Leadership in Vaccinology Expertise (Alive), Faculty of Health Sciences, University of the Witwatersrand, Johannesburg, South Africa; j Department of Regulation and Prequalification, World Health Organization, Geneva, Switzerland

**Keywords:** Maternal health, Neonatal health, Surveillance, Vaccine Safety, Multi-country Studies, Pharmacovigilance

## Abstract

The WHO Global Vaccine Safety Multi-Country Collaboration study on safety in pregnancy aims to estimate the minimum detectable risk for selected perinatal and neonatal outcomes and assess the applicability of standardized case definitions for study outcomes and maternal immunization in low- and middle-income countries (LMICs). This paper documents the operational lessons learned from the study.

A prospective observational study was conducted across 21 hospitals in seven countries. All births occurring at sites were screened to identify select perinatal and neonatal outcomes from May 2019 to August 2020. Up to 100 cases per outcome were recruited to assess the applicability of standardized case definitions. A multi-pronged study quality assurance plan was implemented. The impact of the COVID-19 pandemic on site functioning and project implementation was also assessed.

Multi-layered ethics and administrative approvals, limited clinical documentation, difficulty in identifying outcomes requiring in-hospital follow-up, and poor quality internet connectivity emerged as important barriers to study implementation. Use of electronic platforms, application of a rigorous quality assurance plan with frequent interaction between the central and site teams helped improve data quality. The COVID-19 pandemic disrupted data collection for up to 6 weeks in some sites.

Our study succeeded in establishing an international hospital-based surveillance network for evaluating perinatal and neonatal outcomes using common study protocol and procedures in geographically diverse sites with differing levels of infrastructure, clinical and health-utilization practices. The enhanced surveillance capacity of participating sites shall help support future pharmacovigilance efforts for pregnancy interventions.

## Highlights

• The feasibility and utility of conducting a large multi-country sentinel-site based surveillance study was evaluated in the context of maternal immunization vigilance.
• Prospective observational surveillance was undertaken for seven adverse perinatal and neonatal outcomes across 21 sites in seven countries over a 12-month period May 2019 to August 2020.
• Implementation of this study across geographically diverse sites with differing levels of infrastructure, clinical and health-care utilization practices was affected by several challenges, including, time-consuming ethics and administrative approvals, limited clinical documentation, challenges in follow-up and outcome identification and poor internet connectivity.
• The onset of the COVID-19 pandemic during the active data collection phase of the study also affected study implementation, temporarily disrupting data collection and study monitoring at many sites.
• Use of electronic platforms for data collection and issue resolution and application of a quality assurance plan with frequent interaction between central and site study teams emerged as key facilitators for study operationalization.
• The operational lessons learned from our study provide valuable insights for future benefit-risk assessments of pregnancy interventions and further strengthening vaccine pharmacovigilance in low- and middle-income countries.

## Introduction

Maternal immunization has emerged as a promising intervention for reducing maternal, perinatal and neonatal morbidity and mortality globally [1], [2]. Despite a growing body of evidence regarding their benefits, uptake of maternal immunization, particularly in LMIC settings, has been hindered by lack of data on disease burden, concerns about vaccine safety and effectiveness and low public threshold for acceptance of adverse events following immunization (AEFIs) [3], [4], [5]. Vaccine clinical trials lack the capacity to detect rare and long-term AEFIs and, as a vulnerable group, pregnant women have been historically excluded from them [6], [7]. Post-licensure safety surveillance for pregnancy interventions is complex and requires tracking exposure and maternal, perinatal and neonatal outcomes longitudinally and in a linked fashion [4], [8]. Several outcomes may be assessed as end-points for both benefit and risk evaluations; for instance, stillbirths, which may be reduced over the long-term following maternal immunization, but may also be reported as a potential AEFI in the short-term [8]. Diversity in terminologies, clinical record keeping and healthcare utilization practices affect the reliable identification of outcomes of interest and establishment of baseline rates in LMICs [8], [9].

The Global Vaccine Safety Blueprint calls for large multi-country surveillance studies to clarify complex vaccine safety issues and enhance pharmacovigilance efforts in LMICs [7]. Past multi-country sentinel site-based studies have demonstrated the feasibility and utility of this approach for evaluating vaccine safety [10], [11], [12]. However, implementing large multi-country studies in geographically, socio-demographically diverse settings with varying clinical and record-keeping practices is operationally challenging particularly in regards to site selection, ethical and regulatory approvals and adoption of uniform operating procedures in sites with varying infrastructure [11], [13]. Given the unique challenges, it is important to evaluate this concept in the context of maternal immunization vigilance in resource-constrained settings.

The Global Vaccine Safety Multi-country Collaboration (GVS MCC) project on safety in pregnancy aims to estimate the minimum detectable risk for select perinatal and neonatal outcomes and assess the applicability of standardized Global Alignment of Immunization Safety Assessment (GAIA) case definitions for study select outcomes and maternal immunization in LMICs as part of the WHO Global Vaccine Safety Initiative [14], [15]. The COVID-19 pandemic emerged during the active data collection phase of the study. This publication documents the operational experience of establishing a large multi-country hospital-based surveillance for evaluation of perinatal and neonatal outcomes and examines the impact of the COVID-19 pandemic on the study. The paper sheds light on approaches undertaken to address common challenges in conducting multi-country studies and describes unique challenges encountered in the context of maternal immunization vigilance. As more pregnancy interventions become available, particularly in resource constrained settings lacking robust systems for pharmacovigilance; the operational lessons learned from our study may serve as a useful guide for informing future benefit-risk evaluations.

## Methods

### Study design and setting

A facility based, prospective observational study was conducted in 21 sites across six LMICs and one high-income country over a 12-month period between May 2019 to August 2020. Table 1 describes the study sites and their characteristics. The selected study outcomes were low birthweight, preterm birth, small for gestational age (SGA), stillbirth, in-hospital neonatal death, neonatal infection and postnatally diagnosed congenital microcephaly. As specified by the GAIA case definitions, surveillance was undertaken for three types of neonatal infections: meningitis, invasive bloodstream and respiratory infection. In addition to these outcomes, the applicability of the GAIA case definition for maternal immunization was also assessed. The maternal immunization exposure status, including date and time of vaccination, as well as batch details were collected (when available as part of routine patient documentation) for all vaccines administered in pregnancy and within 30 days prior to the last menstrual period. The process of site selection and network establishment has been described in a previous publication [14].

**Table 1:** Study site characteristics.

Country/Sitename	Type of healthcare	Facility ownership	Record keeping
Ghana			
St Joseph's H	Secondary	Public	Paper
Ejisu H	Secondary	Public	Paper
Tema GH	Secondary	Public	Paper
Eastern RH	Secondary	Public	Combination
United Republic of Tanzania			
Mbeya ZRH	Tertiary	Public	Combination
St Francis RH	Tertiary	Public Private Partnership	Paper
Mbeya RRH	Tertiary	Public	Paper
Zimbabwe			
Mbare PC	Primary	Public	Paper
Mutare PH	Tertiary	Public	Paper
Islamic Republic of Iran			
Mahdieh H	Tertiary	Public	Combination
Shohada TH	Tertiary	Public	Combination
Spain			
Castellon GUH	Tertiary	Public	Electronic
Dr Peset UH	Secondary	Public	Electronic
India			
JSS H	Tertiary	Private	Combination
Grant GMC	Tertiary	Public	Paper
IMS SUM H	Tertiary	Private	Combination
Kasturba MC	Tertiary	Private	Combination
MP Shah MC	Tertiary	Public	Paper
SKIMS	Tertiary	Public	Paper
Nepal			
Patan H	Tertiary	Public	Paper
BP Koirala	Tertiary	Public	Combination

Abbreviations- BP: BP Koirala Institute of Health Sciences; GH: General Hospital; GMC: Government Medical College; GUH: General University Hospital; H: Hospital; IMS SUM: Institute of Medical Science and Sum Hospital; MC: Medical College; PC: Polyclinic; PH: Provincial Hospital; RH: Referral/Regional Hospital; RRH: Regional Referral Hospital; SKIMS: Sher-i-Kashmir Institute of Medical Sciences; TH: Teaching Hospital; UH: University Hospital; ZRH: Zonal Referral Hospital.

For assessing background rates and minimum detectable risk, all study outcomes identified were included in the study. Assuming 50% of all cases would meet the case confirmation criteria, up to 100 cases per outcome were recruited per site to enable estimation of the proportion of cases meeting standardized case definition with a 20% relative precision. To minimize chances for bias, sites were requested to recruit the first two cases identified for each outcome every week.

### Study management and scientific oversight

**Fig. 1:**
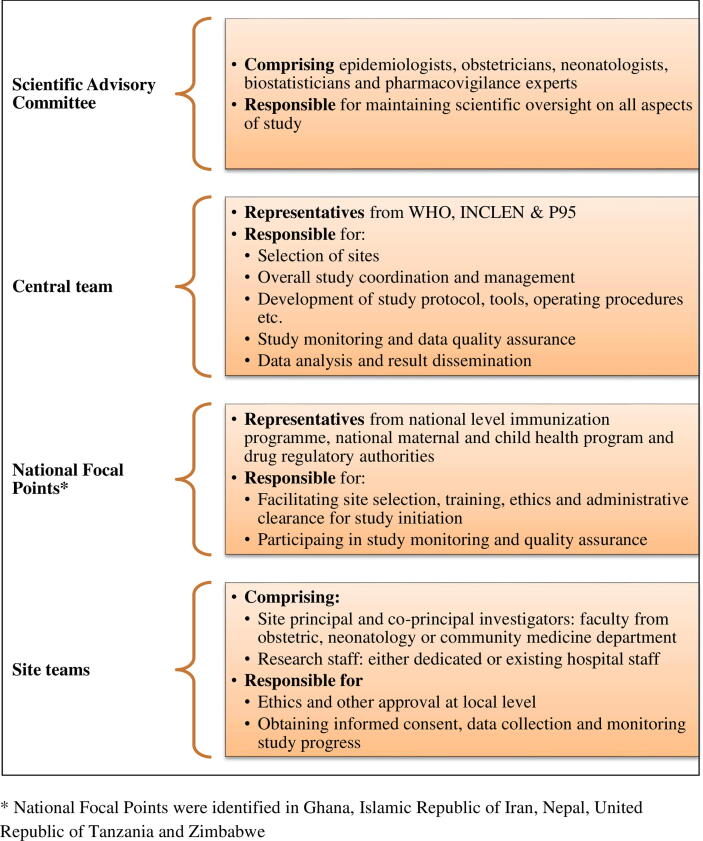
Study management and scientific oversight.

Fig. 1 describes the multi-level mechanisms for study management and scientific oversight. A diverse team of epidemiologists, biostatisticians, information technology (IT) and pharmacovigilance experts coordinated the study at the central level, while faculty members and research staff implemented the study at the site-level. Based on findings from a previous proof-of-concept multi-country collaboration study [10], National Focal Points (NFPs) were identified to facilitate study implementation in some countries. A seven member advisory committee maintained scientific oversight of the study.

### Protocol development, ethics and administrative approvals

The study protocol was developed in consensus with participating sites over a two-day investigator meeting in 2017. In addition to the study protocol, the requirements for national and local administrative clearances and training needs necessary for study initiation were discussed in detail among the central team, site investigators, and NFPs attending the investigator meeting. The master protocol was submitted to the WHO Ethics Review Committee (ERC) and by each participating site to local, national, or independent Ethics Committee (EC) as applicable. Additional regulatory clearances such as data transfer agreements and administrative approvals were sought based on country or site-level regulations and norms.

### Training and study support documents

Two to three-day country level workshops were conducted in-person using standardized training materials and support documents, such as the study standard operating procedures (SOP) and software application manuals. Feedback from each training was used to improve materials for subsequent trainings; for instance, a handy aide-mémoire was developed based on observed challenges in understanding certain aspects of the study. Following the workshop, sites completed a simulation exercise providing hands-on experience on study SOPs and data entry procedures. The central team provided additional training based on site performance during the simulation exercise.

### Data collection tools and procedures

**Fig. 2:**
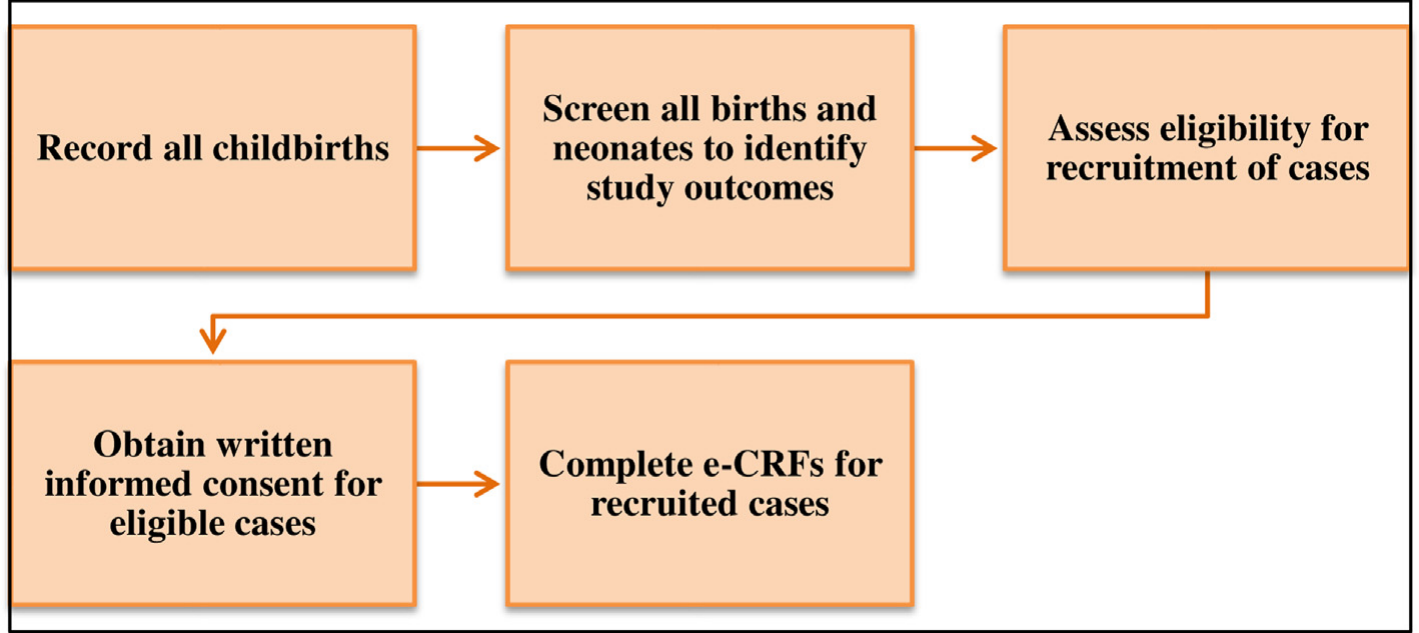
Study data collection process.

An android-based application, SOMAARTH III [16], was developed in-house at INCLEN and was used to implement study procedures and data collection. The application and electronic case report forms (e-CRFs) were tested internally by the study team and piloted at one study site prior to finalization. The SOMAARTH III application has built-in alerts for missing and invalid data, range-checks, automated skip logic, role-based access control, and dynamic form activation to minimize errors. The software allotted an auto-generated unique identification number to each registered birth, enabling tracking of mother–child dyads in a linked manner during the study. Study data could be collected and stored in offline mode, and only required internet connectivity for data submission. To aid assessment of eligibility for recruitment, a report module was introduced post study initiation for tracking the number of childbirths, and study outcomes recorded on a weekly basis. The data collection process is summarized in Fig. 2.

### Data management, quality assurance & monitoring

**Fig. 3:**
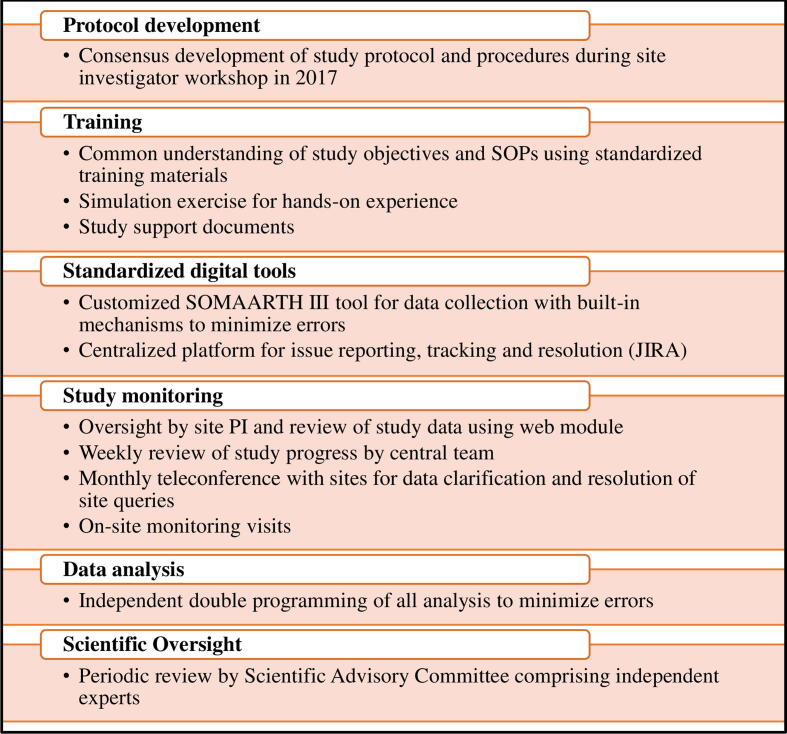
Multi-pronged quality assurance mechanism.

A data management plan was developed specifying the procedures for data collection, management, safety, privacy, sharing and archiving. A multi-pronged study quality assurance plan was implemented to ensure collection of quality data during the study (Fig. 3).

Data were reviewed remotely on a periodic basis to evaluate adherence to study protocol, data quality, and medical congruency by the central study team. Standardized templates and algorithms were developed using Stata version 15.1 [17] to automate data reviews. Discrepancies identified during the review were discussed and clarified with sites over teleconferences (held fortnightly in the first month and monthly thereafter). A mid-course country level call was also conducted with participation from all site teams and NFPs to review study progress, share experiences and discuss challenges. A centralized platform (JIRA®) was adopted for query generation, resolution, tracking and documentation [18].

In addition to remote monitoring, on-site visits were planned, initially to at-least 4 sites, to check compliance with study protocol and SOPs. A standardized template was developed to facilitate systematic conduct of on-site monitoring. All visiting team members were apprised of the objectives, provided a site performance report and a randomly chosen selection of recruited cases for comparison with source documents and validation. After each visit, a detailed report was prepared and discussed with the sites.

Periodic feedback was sought from the scientific advisory committee. A quarterly newsletter was circulated to the sites, NFPs, and scientific committee to keep them informed about the status of the study.

Independent, double programming of analysis was undertaken based on a pre-specified statistical analysis plan using R version 3.6.0 [19] and Stata version 15.1 [17]. Results from the double programming were matched and discrepancies identified were discussed and resolved.

### Impact of COVID-19

To identify and potentially address pandemic related challenges additional teleconferences were conducted with sites. Upon completion of data collection, sites were asked to complete a questionnaire aiming to understand the impact of the pandemic on study procedures and health care at study sites.

## Results

Epidemiological findings from the study can be accessed from the study report [20]. Key barriers and facilitating factors identified during various stages of study implementation are described below.

### Protocol development, ethics and administrative approval

The study protocol was refined significantly based on feedback obtained from the investigator workshop, including switching from retrospective to prospective study design and finalization of study outcomes under surveillance.

Starting in November 2018, the master study protocol was approved by the WHO ERC; the national EC in Ghana, the United Republic of Tanzania, Zimbabwe; the institutional ECs in all sites in India; local ECs for the two sites in Nepal and some sites in Zimbabwe and the United Republic of Tanzania; the regional EC for Spain sites and an independent EC for sites from the Islamic Republic of Iran sites. Additional clearance was obtained from the National Health Research Council in Nepal. Despite prior planning and discussion at the investigator meeting, the time for obtaining ethics and regulatory approvals tended to be unpredictable and highly variable across sites (particularly for ECs that did not meet frequently or were suddenly dissolved). This process took between one and seven months across sites and approval was not uniform; in Spain the EC did not approve the collection of any individual-level data without informed consent, therefore only the aggregated number of births and study outcomes per month were recorded. While the initial feasibility assessment identified 24 eligible sites for participation in the study [14], data collection could not be initiated at three sites due to challenges in receiving ethics and administrative approvals in time.

### Study management and scientific oversight

A team of over 15 members at the central level and 80 members at the site-level supported the study implementation. The NFPs facilitated site selection, training, ethical and administrative clearances for study initiation in many countries and in some countries helped streamline the review process. Advice from the scientific advisory committee helped address emerging challenges during the course of the study and provided an opportunity for course-correction.

### Training and study support documents

**Fig. 4:**
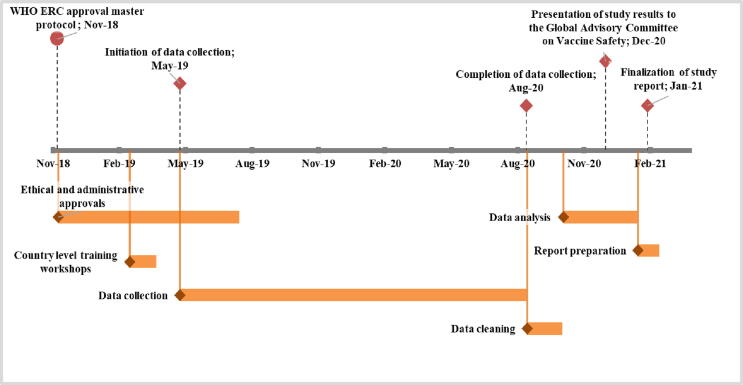
Study timelines and milestones.

Training workshops were completed over a 3-month period starting in February 2019 and data collection was initiated at the final site by July 2019 (Fig. 4). Although planning for network operationalization was time and labour intensive, the implementation of a rigorous training programme and provision of simulation exercise and supportive documents promoted a thorough understanding of the study. However, certain aspects of the SOP and data entry application (e.g. choice of single vs. multiple response options) remained susceptible to frequent errors. Results of the simulation exercise post-training underscored the need for additional training at some sites; as a mitigation measure, audio-visual training materials were prepared to promote self-learning and facilitate training of new staff at sites during the course of the study.

### Site-level preparation and support for study implementation

Allocation of dedicated resources (infrastructural, technological, and human resource related) was an important factor in streamlining day-to-day study operations at sites. Early on in the study, non-availability of dedicated study staff resulted in substantial delays in data entry that ultimately led to study termination in one site. Another site reported challenges in accessing patient records due to lack of inter-departmental support for the study. This, in addition to the onset of the COVID-19 pandemic resulted in early termination of data collection at the site at 8 months. Additionally at one site, data collection was interrupted for nearly 2 months as the study staff had additional responsibilities that prevented timely data collection and submission for the study. The issue was resolved once additional support staff were allocated to the study team. Difficulty in retrieving patient records from the medical records department (MRDs) added to the complexity of collecting large amounts of data in a timely manner, particularly at sites with heavy patient-load.

### Electronic tools for data collection and query resolution

Electronic study tools like the SOMAARTH III and JIRA® applications played a vital role in assuring data quality. However, the lag between collection and submission of data emerged as an important barrier in adherence to study SOPs and timely review of data. The use of multiple software programmes added to the complexity of the study procedures. In some instances, lack of access to quality, uninterrupted network prevented optimal utilization of study applications and obstructed monthly review.

### Data collection

The absence of quality information, either due to difficulty in retrieval of information (e.g. non-availability of maternal immunization card at the time of case identification) or due to absence of routine clinical documentation (e.g. for head circumference measurements), hindered outcome identification and classification. Outcomes such as SGA and congenital microcephaly, which were dependent on chart-based algorithms for confirmation, were underreported. In contrast to outcomes identifiable at birth such as low birthweight and preterm birth, the requirement of linking maternal and neonatal medical records and in-hospital follow-up posed a challenge to the identification of neonatal infection and death cases. The use of multiple data sources (hospital registers, patient case records) resulted in data discrepancies for key elements such as birthweight and gestational age and contributed to misclassification of some cases. The eligibility criteria for recruitment (first 2 cases for each outcome per week) was difficult to track and sites often over- or under-recruited cases for the week. To counter underreporting and simplify study procedures by allowing consecutive recruitment of cases, the study protocol was further amended (version 3.0: November 1, 2019).

Maternal vaccination data was not routinely recorded in patient case records and even in instances where ANC cards were available at the time of hospitalization, the information lacked the granularity necessary for systematic investigation of AEFIs; the batch number, brand, and time of maternal vaccination could only be ascertained in two sites.

### Data quality assurance

Concurrent monitoring of study data helped detect data discrepancies and provided an opportunity for mid-course correction. Over 3000 queries were generated, and 226 teleconferences were conducted to discuss and resolve these queries. Some common errors identified included discrepancies in birthweight and gestational age data, missed outcome identification and misclassification of cases. Sites where large discrepancies were identified were subsequently asked to review patient medical records from study inception and report any additional missed outcomes separately. The use of web-module for site-level data review was limited and many site PIs developed independent mechanisms, such as weekly meetings with study staff, for reviewing study progress and data quality at the site-level.

Challenges identified during remote monitoring necessitated more on-site monitoring visits than originally planned. On-site monitoring visits were completed at least once in 19 of the 21 selected study sites and multiple visits were performed in four sites due to persistent non-compliance to SOPs and errors in data collection and entry. Site visits could not be conducted at two sites due to logistical challenges. While members of the central team conducted most site visits, the site visits in Zimbabwe and one site in the United Republic of Tanzania were completed by the NFPs. To promote cross-learning, site investigators were also invited to be part of the visiting team in some sites in the United Republic of Tanzania and Ghana.

A key finding of the on-site monitoring visits pertained to issues in the informed consent process across multiple sites; between 0 and 30% of the ICF reviewed were missing across sites and at least 2 sites, none of ICFs reviewed were duly signed. These issues were reported to the WHO ERC and assessed to arise likely from poor record keeping, rather than misconduct. The WHO ERC adjudged that the ethical risks of keeping data on such subjects were low; on the contrary, removing such data would have affected either the study objectives or the validity of the study, by overstating the quality of the record keeping.

### Impact of COVID-19

The COVID-19 pandemic started while data collection for the study was ongoing. Most participating health facilities (n = 15) reported that COVID-19 cases were managed at their sites, whereas at 5 sites COVID-19 cases were referred elsewhere and one site reported no COVID-19 cases during the study period. Seventeen sites tested for SARS-CoV-2; mothers and neonates tested positive at 11 and 4 sites respectively. Nearly half of the sites (n = 9) identified pandemic associated restrictions as a hindrance to quality maternal care. Many sites indicated that women went elsewhere to give birth for a variety of reasons including lack of transport, lack of staff and fear of nosocomial COVID-19.

The pandemic placed additional demands on the site investigation teams and disrupted data collection for up to 6 weeks at some sites. Despite these disruptions, most sites (n = 17) indicated that the standard study procedures were not compromised. Few sites (n = 4) adapted study SOPs to enable project implementation, e.g. shifting to virtual site-level site meetings instead of regular in-person meetings to monitor study progress. Supplementary table S1 describes the key findings from the COVID-19 impact assessment questionnaire in further detail.

## Discussion

Our study succeeded in establishing an international hospital-based surveillance network for evaluating perinatal and neonatal outcomes using a common study protocol and procedures, in geographically diverse sites with varying infrastructure, clinical and healthcare utilization practices. Although our study focused on maternal immunization vigilance, the operational lessons learned may be relevant to a wide range of multi-country or multi-site collaborative studies.

Time-consuming and complicated processes for ethics and administrative clearance delayed study initiation and highlighted the need for streamlining of the approval process at national and local levels. Dependence on support from multiple specialities and departments for data collection affected its quality and timeliness at some sites. In particular, retrieval of patient records from the Medical Records Department (MRD) was challenging at many sites. Future safety studies will benefit from allocation of dedicated study staff for study implementation and building mechanisms for ensuring cross-specialty and inter-sectoral support for the study at the site and national levels.

The systematic preparations undertaken for training and use of dynamic electronic applications for data entry and issue management helped minimize data entry errors and protocol deviations. Future studies may benefit from investing in development of customized software that integrates data collection, monitoring and query resolution functions thereby reducing the complexity of using multiple software programmes. Development of additional, more interactive modules and user-generated reports will enhance site-level data quality assurance and monitoring. Allocation of time and resources for more rigorous pilot testing of study CRFs and tools prior to study start and ensuring seamless internet connectivity will improve software utilization and minimize the lag observed between data collection and submission in our study. Investigator motivation and heavy patient-load emerged as additional key site-level factors affecting study implementation and quality of data collected.

The development and implementation of a rigorous study monitoring with frequent opportunities for interaction between the central and site investigator teams supported capacity building for public health surveillance capacity at sites. Despite standardized training, provision of multiple support documents and detailed feedback in the simulation exercise, common issues with SOP adherence and data entry errors occurred across sites. The on-site monitoring visits highlighted issues, which could not be identified during remote monitoring (such as issues with the informed consent process) and provided additional opportunities for clarification of study protocol, SOPs, and data related issues. In particular, the lack of understanding regarding the importance of documenting consent and the need to complete large number of ICFs contributed to the significant challenges regarding informed consent identified in our study. Future studies will benefit from including more interactive training modules dedicated to ethics and informed consent. Provisions for on-site initiation visits by the central study team, as well as periodic refresher trainings may provide additional opportunities for guidance and minimize these errors. Engaging representatives from national level immunization programme, national maternal and child health program and drug regulatory authorities facilitated study implementation in some countries. Early engagement and training of NFPs for on-site monitoring visits will help mitigate the challenges identified in our study and allow closer monitoring of the sites from study inception.

As observed in previous studies, the use of multiple data sources and dependence on diverse charts and algorithms for outcome identification contributed to underreporting of certain outcomes and misclassification of cases in our study [21], [22]. Along with standardization of case definitions, harmonization of common data elements and assessment methods is needed to improve outcome identification and generate reliable baseline rates of maternal, perinatal, and neonatal outcomes. The observational nature of our study may have limited the ability to collect specific information (e.g. maternal immunization or first trimester ultrasound reports) [23]. Future safety studies may benefit from more rigorous outreach to retrieve maternal immunization data and systematic investments are needed to develop digital systems that link maternal vaccination information with health records of pregnant women and their children in LMICs. Findings from our study underscore the need for stronger advocacy among health programme managers and policy makers towards the increased digitization of antenatal cards and their expansion to include additional fields relevant to maternal immunization vigilance. Increasing awareness among neonatologists, obstetricians and adult physicians will also help improve documentation of maternal immunization exposure status in routine clinical practice and programme delivery; thereby enhancing the quality of exposure information available for future studies.

The COVID-19 pandemic temporarily disrupted data collection in our study and may have affected outcome identification at sites. Several sites reported that the pandemic restricted access of pregnant women to ANC and obstetrics services, while others reported an increase in referral of complicated cases of pregnancy during the pandemic. Future studies will benefit from the development and periodic assessment of risk management plans to rapidly identify and address emerging challenges to study implementation.

The lessons learned from our study underscore the need for fostering greater collaboration between the maternal, neonatal and child health, immunization and pharmacovigilance programmes and building unified systems for monitoring outcomes relevant to multiple fields of public health. This is particularly relevant while more and more countries are adopting policy recommendation for COVID-19 vaccination in pregnant women [24]. The enhanced surveillance capacity of participating sites and the operational lessons learned from our study should help support ongoing efforts to strengthen pharmacovigilance capacity in low- and middle-income countries.

## CRediT authorship contribution statement

**Apoorva Sharan:** Conceptualization, Investigation, Methodology, Visualization, Writing – original draft, Writing – review & editing. **Shubhashri Jahagirdar:** Investigation, Methodology, Formal analysis, Writing – review & editing. **Anke L Stuurman:** Conceptualization, Investigation, Methodology, Formal analysis, Writing – review & editing. **Varalakshmi Elango:** Investigation, Project administration, Methodology, Writing – review & editing. **Margarita Riera-Montes:** Conceptualization, Investigation, Supervision, Resources, Methodology, Writing – review & editing. **Neeraj Kumar Kashyap:** Software, Data curation, Writing – review & editing. **Narendra Kumar Arora:** Conceptualization, Methodology, Resources, Writing – review & editing. **Mathews Mathai:** Conceptualization, Methodology, Writing – review & editing. **Punam Mangtani:** Conceptualization, Methodology, Writing – review & editing. **Hugo Devlieger:** Conceptualization, Methodology, Writing – review & editing. **Steven Anderson:** Conceptualization, Methodology, Writing – review & editing. **Barbee Whitaker:** Conceptualization, Methodology, Writing – review & editing. **Hui-Lee Wong:** Methodology, Writing – review & editing. **Clare L Cutland:** Conceptualization, Methodology, Writing – review & editing. **Christine Guillard Maure:** Conceptualization, Funding acquisition, Project administration, Supervision, Writing – review & editing.

## Declaration of Competing Interest

The authors declare that they have no known competing financial interests or personal relationships that could have appeared to influence the work reported in this paper.

## Appendix WHO Global Vaccine Safety Multi-Country Collaboration Sites

Joseph HK Donkor, Tema General Hospital, Tema, Ghana;

Richard Wodah-Seme, St. Joseph’s Hospital, Jirapa, Ghana;

Kwasi Baffour Gyimah, Ghana Health Service: Ejisu Government Hospital, Ejisu, Ghana.

Seth Twum, Eastern Regional Hospital, Ghana;

Issa Sabi, National Institute for Medical Research (NIMR), Mbeya Medical Research Center, Mbeya, United Republic of Tanzania;

Rebecca Mokeha, Mbeya Zonal Referral Hospital, Mbeya, United Republic of Tanzania;

Elias Kweyamba and Henry Marique, St. Francis Referral Hospital, Ifakara, United Republic of Tanzania;

Ismail Macha, Mbeya Regional Referral Hospital, Mbeya, United Republic of Tanzania;

Jaensch Masanga Mutede, Mutare Provincial Hospital, Ministry of Health and Child Care, Zimbabwe;

Prosper Chonzi, City of Harare, Health Department, Ministry of Health and Child Care (for Mbare Provincial Hospital), Zimbabwe;

Maryam Shariati and Elahe Rastkar Mehrabani, Clinical Research Development Center, Mahdiyeh Educational hospital, Shahid Beheshti University of Medical Sciences, Tehran, Islamic Republic of Iran;

Alejandro Orrico-Sánchez, Antonio Carmona and Dafina Petrova, Vaccine Research Department, Fundación para el Fomento de la Investigación Sanitaria y Biomédica de la Comunitat Valenciana, FISABIO-Public Health, Valencia, Spain;

Leslie Lewis, Muralidhar Pai, Shyamla G, Jyothi Shetty, Akhila Hebbar, Sripad Hebbar and Prathap Kumar, Kasturba Medical College, Manipal Academy of Higher Education, Manipal, India;

Bhadresh R Vyas, MP Shah Government Medical College, Jamnagar, India;

Lalit Sankhe, Department of Community Medicine, Grant Medical College, Mumbai, India;

Rachita Sarangi and Jagdish Prasad Sahoo, Indian Institute of Medical Sciences and SUM hospital, Bhubaneswar, Orissa, India;

M D Ravi and H V Prajwala, JSS Academy of Higher Education and Research, Mysuru, Karnataka, India;

Javeed Iqbal Bhat and Bashir Ahmad Charoo, Department of Pediatrics, Sher-i-Kashmir Institute of Medical Sciences Srinagar Jammu & Kashmir, India;

Nisha Keshary Bhatta, Shyam Prasad Kafle, Mukesh Bhatta, Mohan Chandra Regmi, B.P.Koirala Institute of Health Sciences, Dharan, Nepal;

Prerana Kansakar and Ganesh Shah, Department of Pediatrics, Patan Academy of Health Sciences, Nepal.
