# Supplementary material for: Operational lessons learned in conducting an international study on pharmacovigilance in pregnancy in resource-constrained settings: The WHO Global Vaccine safety Multi-Country collaboration project
Source: Vaccine X. 2022 Apr 9;11:100160. doi: 10.1016/j.jvacx.2022.100160 (PMC8993756; doi:10.1016/j.jvacx.2022.100160)
Supplement: Supplementary data 1 [file mmc1.docx]

**Supplementary table S1: COVID-19 impact assessment**

| Domain | Questions | Summary response from sites |
| --- | --- | --- |
| Impact on general site functioning | When was the COVID-19 outbreak declared in your country? | - Earliest date: 19^th^ February 2020; two sites in Iran (Islamic Republic of) - Latest date: 30^th^ March 2020, one site in Zimbabwe |
|  | Were COVID-19 patients managed in your facility or referred elsewhere? | - At facility in 15 sites and referred at an additional 5. - One site reported no COVID-19 cases at facility |
|  | Did your facility do testing or diagnosis for COVID-19? | - Yes at 17 of the 21 sites |
| Impact on obstetric & neonatal care | Were any mothers or neonates diagnosed with COVID-19 while they were at the facility? | - Yes at 11 sites - Between 1-40 mothers diagnosed with COVID-19 at sites - One site reported that a mother died from COVID-19 related complications whilst in labour - Between 0-40 neonates diagnosed with COVID-19 whilst at site |
|  | Did any restrictions imposed because of the pandemic affect the ability of mothers to access health care for childbirth, or for care to be sought for neonates? | - 9 sites reported that restrictions imposed because of the pandemic affected the ability of mothers to access healthcare for childbirth - Many sites indicated women went elsewhere to give birth (lack transport, lack of staff, fear of nosocomial COVID19) - Regular ANC was affected at 10 sites |
|  | In your opinion, did the characteristics of mothers delivering at your facility change during the COVID pandemic? | - 8 sites reported a perceived change in characteristics of mothers (this was not supported by study data) - Sites noted an increase in proportion of high-risk mothers visiting the site for childbirth |
| Impact on study procedures | Please explain the main effects that restrictions had on staff involved in the study | - 7 sites reported that the pandemic restricted movement and affected the ability of study staff to work |
|  | Please explain the main effects that pandemic on study procedures at the site | - 4 sites reported that they shifted from in-person to virtual monitoring and quality checks - At two sites, data collection was halted for nearly 6 weeks |
